# Identification of Low-Confidence Regions in the Pig Reference Genome (Sscrofa10.2)

**DOI:** 10.3389/fgene.2015.00338

**Published:** 2015-11-27

**Authors:** Amanda Warr, Christelle Robert, David Hume, Alan L. Archibald, Nader Deeb, Mick Watson

**Affiliations:** ^1^Division of Genetics and Genomics, The Roslin Institute and Royal (Dick) School of Veterinary Studies, University of EdinburghEdinburgh, UK; ^2^Genus plc., HendersonvilleTN, USA

**Keywords:** missassembly, copy number variable regions, structural variation, draft assemblies, false positives

## Abstract

Many applications of high throughput sequencing rely on the availability of an accurate reference genome. Variant calling often produces large data sets that cannot be realistically validated and which may contain large numbers of false-positives. Errors in the reference assembly increase the number of false-positives. While resources are available to aid in the filtering of variants from human data, for other species these do not yet exist and strict filtering techniques must be employed which are more likely to exclude true-positives. This work assesses the accuracy of the pig reference genome (Sscrofa10.2) using whole genome sequencing reads from the Duroc sow whose genome the assembly was based on. Indicators of structural variation including high regional coverage, unexpected insert sizes, improper pairing and homozygous variants were used to identify low quality (LQ) regions of the assembly. Low coverage (LC) regions were also identified and analyzed separately. The LQ regions covered 13.85% of the genome, the LC regions covered 26.6% of the genome and combined (LQLC) they covered 33.07% of the genome. Over half of dbSNP variants were located in the LQLC regions. Of copy number variable regions identified in a previous study, 86.3% were located in the LQLC regions. The regions were also enriched for gene predictions from RNA-seq data with 42.98% falling in the LQLC regions. Excluding variants in the LQ, LC, or LQLC from future analyses will help reduce the number of false-positive variant calls. Researchers using WGS data should be aware that the current pig reference genome does not give an accurate representation of the copy number of alleles in the original Duroc sow’s genome.

## Introduction

Contemporary genetics research benefits from genomics tools and resources, including DNA sequencing and single nucleotide polymorphism (SNP) chips, which facilitate detailed quantitative molecular characterization of genetic variation at the population and individual level. A high quality reference genome sequence for the species of interest is an invaluable asset for the discovery of molecular genetic variants. Most reference genome sequences for species with large, complex genomes are incomplete representations of the genome sequence of a single individual or a small number of individuals. Given the extent of insertion/deletion (indel) polymorphisms and copy number variation (CNV) within species, such individual reference genomes do not contain all the sequences present in the species of interest. Thus, there are two major flaws in the current single linear model for reference genomes as a framework for discovery and analysis of genetic variation: (1) errors and gaps in the reference genome assemblies most of which are incomplete drafts; and (2) using a haploid genome of one individual to represent the genome(s) of a species. In this paper we focus solely on the former.

Studies that employ variant calling from sequencing data to find variation in the genome produce large variant call sets (Robert et al., 2014; Belkadi et al., 2015; Bianco et al., 2015; Gudbjartsson et al., 2015). Most of these calls will be either false-positive, not relevant to the phenotype under investigation or benign (MacArthur et al., 2012). Failure to detect true variants (i.e., false-negatives) will also occur either as a result of insufficient sequence depth or gaps in the reference genome (real or technical). Filtering these datasets reduces the number of variants to a level which can be validated, however, in the process researchers risk discarding the variants they are looking for.

Many applications of high throughput sequencing rely heavily on the accuracy of the available reference genome for the species. Errors in the reference genome increase the number of false-positive variant calls in data, resulting in a need for more stringent filters which may increase the risk of removing true-positives. Shortcomings in the reference genome will also increase the risk of missing true variants (i.e., false-negatives). The human genome is more accurate than that of many other species and more resources are available to aid in the filtering of false-positive variants. Many reference genomes have a draft status and gaps and misassemblies are not uncommon (Kelley and Salzberg, 2010). Identifying misassembled regions in the reference genomes of non-human species and excluding them from analysis will help to reduce false-positives in variant calling data.

Whole genome sequencing (WGS) produces fairly consistent coverage across the genome (Belkadi et al., 2015), however, the PCR step in the Illumina library preparation pipeline is known to introduce bias, particularly in regions of high or low GC content (Kozarewa et al., 2009). Modifications have been introduced to protocols to reduce this bias (Aird et al., 2011), however, sequencing depth and quality in GC-rich and -poor regions remain unreliable when using protocols involving a PCR step. Previous work has shown that CNV can be accurately detected in WGS data by looking for areas of excessively high or low read counts following adjustment for GC content (Yoon et al., 2009; Zhang and Backstrom, 2014). To identify misassemblies in the chicken genome, a previous study used a pool of multiple birds to account for true variation between individuals, treating regions where all individuals show low read counts as false tandem duplications (Zhang and Backstrom, 2014).

In this paper, we look to identify low-confidence regions in the reference genome assembly Sscrofa10.2 using WGS reads from T. J. Tabasco (Duroc 2-14), the Duroc sow whose DNA was used in the assembly (Groenen et al., 2012). The assembly was constructed using a BAC-by-BAC method, covers 18 autosomes and 2 allosomes (with the Y chromosome constructed separately from the DNA of male pigs), and contains many gaps and sequences on unplaced scaffolds. Ideally, an individual’s sequencing reads mapped to that individual’s own assembled genome would show no true structural variation and any areas of structural variation could be considered a misassembly. But the reference genome is a haploid representation and cannot reflect areas of true heterozygous structural variation accurately. However, a conservative approach would treat variant calls in these areas as low-confidence until further verified. Regions with no structural variation between the sequencing reads and the reference genome can be considered high-confidence.

In addition to using coverage to detect potential duplications or collapses, we use other indicators to identify different kinds of structural variation such as inversions, deletions and insertions as has been done previously to identify potentially disease causing structural variation in human genomes (Tuzun et al., 2005). Illumina paired-end sequencing generates read-pairs from the same DNA fragment that are a known distance apart (usually following a normal distribution), and in a known orientation with respect to the reference genome. Therefore, when read pairs are mapped to a reference, if they are not in the expected orientation, or are an abnormal distance apart, this may also be an indication of errors in the assembly.

Finally, when mapping reads from the same animal to the reference genome created from that animal, there should be no homozygous variant calls.

In this work, regions with abnormally high or low coverage (LC), with high proportions of reads with unexpected insert sizes or a high proportion of reads which were improperly paired were identified. In addition, SNP and indel calling was carried out. Regions were considered low quality (LQ) if they had high coverage, a high proportion of unexpected insert sizes or improperly paired reads or if they were in proximity to a homozygous variant. LQ regions are the most likely to represent misassemblies in the genome. Regions which had LC were analyzed separately; these regions may not necessarily be misassembled, but have poor coverage and may therefore be unreliable for accurate variant calling. Both regions were also analyzed together in a combined dataset (LQLC).

Following identification of regions of the reference which may be unreliable, publicly available data sets were downloaded and overlap with the regions calculated. The data sets downloaded were the coding region, dbSNP variants, copy number variable regions (CNVRs) identified by Paudel et al. (2013) using a method that assesses read depth, and gene predictions based on data obtained using RNA-seq methods. These data sets allowed for identification of the proportion of the coding region overlapping the unreliable regions, and to assess how commonly used methods of SNP and indel calling, CNVR calling and RNA-seq may have been affected by unreliable regions of the genome assembly. We would expect the coding region to be under represented in the LQLC regions because the coding region is generally more complex, which should make assembly more accurate. If the unreliable regions are enriched for calls in these datasets, it may suggest that analysis of these regions produces a higher level of false-positives than the rest of the genome.

## Materials and Methods

### Sample, Sequencing, and Alignment

Eight sets of paired-end, whole-genome, Illumina sequencing reads from a single sample from T. J. Tabasco, the sow whose genome was used to construct Sscrofa10.2, were used in this study^1^. BWA (v0.6.2: Li and Durbin, 2009) was used to align the reads to the Sscrofa10.2 reference assembly using default parameters. The reads were mapped to both the chromosomes and the unplaced scaffolds from the assembly. Any reads which mapped to chromosome Y were excluded as the sequences were from a female pig; consequently, we are unable to comment on the quality of the assembly of chromosome Y.

### Identifying Regions with Abnormal Coverage

SAMtools was used to filter the data to remove reads with a mapping quality less than 2 or which were improperly paired. BEDtools (v2.16.2: Quinlan and Hall, 2010) bamtobed was used to extract the chromosome, start positions and the end positions of whole sequencing fragments. BEDtools GenomeCov was then used to find per-base fragment coverage across the genome. BEDtools MakeWindows was used to make windows of 1000bp across the whole genome. Gap data was downloaded from the UCSC table browser (Karolchik et al., 2004) and BEDtools intersect was used to remove windows intersecting gap data. The median coverage for each 1000 base window across the genome was calculated. GC content is known to have a significant effect on coverage in sequencing methods that involve a PCR stage (Kozarewa et al., 2009). Coverage was normalized by GC content as described by Yoon et al. (2009). Briefly, the read coverage in each 1 Kb window (w) was adjusted by a multiplying factor *f*, with *f* equal to the ratio of the overall median across all windows divided by the median of all windows with the same GC percentage as that of the window *w*. Using the median instead of the mean prevented these values from being inflated by extreme outliers, as described by Zhang and Backstrom (2014). Any window with a normalized coverage over 55 or under 27 (2 *SD* from the mean; 41) was defined as having an abnormal coverage.

The removal of multimappers prior to coverage analysis may cause the detection of LC regions in certain sequence contexts in the genome that are more likely to contain multimappers (e.g., repetitive regions). Multimapped reads were extracted from the original bam file and read counts for these were calculated using Bedtools Coverage and the same 1000 bp windows used in the above coverage analysis; additionally raw read counts for each window were calculated in the same way from the original bam file. The percentage of reads in each window which were multimapped was calculated. Windows with >50% multimapped reads are likely to have been identified as LC due to the removal of these reads before coverage analysis. The regions with >50% multimappers were merged and intersect with the LC regions was calculated using Bedtools.

### Identifying Regions with Abnormal Insert Sizes

The mean and standard deviation of the insert sizes was calculated using Picard InsertSizeMetrics ^2^ (v1.113). Insert sizes were considered abnormal if they were more than 2 *SD* from the mean (427 bp). The merged BAM file was filtered for abnormally large (above 588 bp) and small (below 266 bp) insert sizes. BEDtools coverage was used to find the read count of the abnormal reads and the original BAM file using 1000 base windows with 200 overlap created with BEDtools MakeWindows. These data were used to calculate the percentage of abnormal reads in each window. A high proportion of small insert sizes was defined as a window with over 9.47% small insert sizes (2 *SD* above the mean of 4.22%) and a high proportion of large insert sizes was defined as a window with over 1.86% large insert sizes (2 *SD* above the mean of 0.12%).

### Identifying Regions with a Low Proportion of Properly Paired Reads

The mapped reads were filtered using SAMtools for the SAM flag 0×2, removing reads which were flagged as improperly paired. The percentage of properly paired reads was calculated as described for insert sizes. Any window with fewer than 70.59% (2 *SD* below the mean of 92.5%) properly paired reads was considered abnormal.

### Variant Calling

Single nucleotide polymorphism and indels were called using SAMtools mpileup, BCFtools and vcfutils varFilter. The resultant vcf file was filtered for homozygous variants, indicative of errors in the reference genome or sequencing errors. In order to include the entire regions covered by reads overlapping each variant, the regions spanning from 100 bases before to 100 bases after each variant were considered low quality.

### Merging

BEDtools was used to merge the regions identified by the above parameters into LQ, LC, and LQLC regions. BEDtools intersect was used to find regions of each group which overlapped with the coding region (regions downloaded from UCSC table browser; Karolchik et al., 2004). Sanger’s gEVAL website^3^ was used to inspect BAC and fosmid end alignments in a number of the identified regions.

### Assessing Effect of Identified Regions on Public Data

Known variant data were downloaded from dbSNP (Sherry et al., 2001) and the number of variants overlapping the abnormal regions were calculated. To assess the potential effect of these regions on WGS resequencing studies in pigs, the regions identified as CNVRs in Paudel et al. (2013) were downloaded and the number of regions overlapping the abnormal regions from the current study were calculated. Gene predictions based on RNA-seq data were downloaded from Ensembl (Cunningham et al., 2015) and the number of bases overlapping the identified regions calculated.

## Results

### Alignment

582,271,856 reads mapped to the reference and 94.66% of these were properly paired (551,173,366 reads).

### Abnormal Regions

**FIGURE 1:**
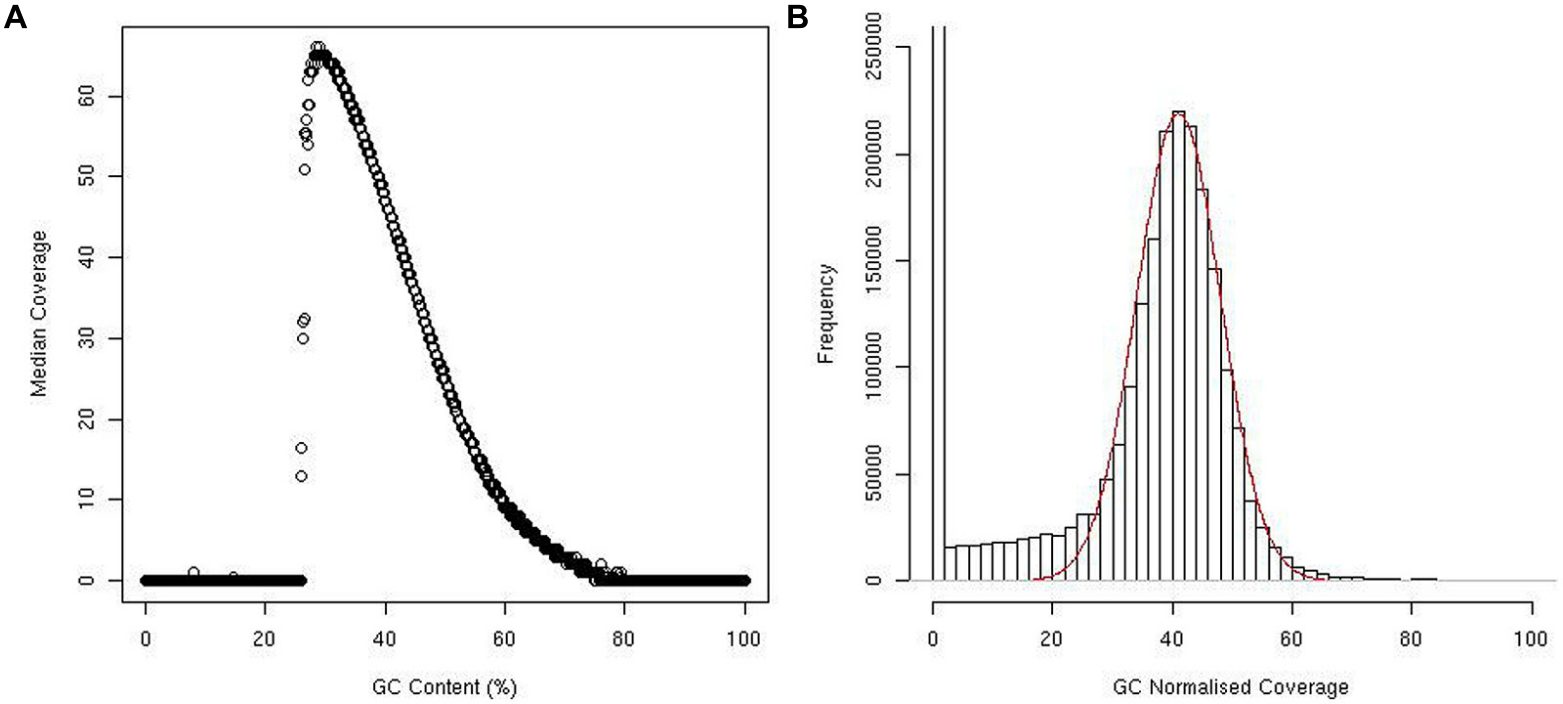
**Plot showing median coverage of windows against percentage of GC content **(A)**.** Histogram showing the distribution of window coverage, red line represents a normal distribution **(B)**.

The effect of GC content on median coverage was as expected, with both high and low GC content regions having poor median coverage (**Figure 1A**).

While the coverage following GC normalization did follow a normal distribution, several extreme outliers inflated the mean and standard deviation. R (R Development Core Team, 2009) was used to find the mean and standard deviation of the majority of the data by overlaying a normal distribution on the data (**Figure 1B**). Using this method, we determined the mean coverage to be 41X and the standard deviation to be 7.

**FIGURE 2:**
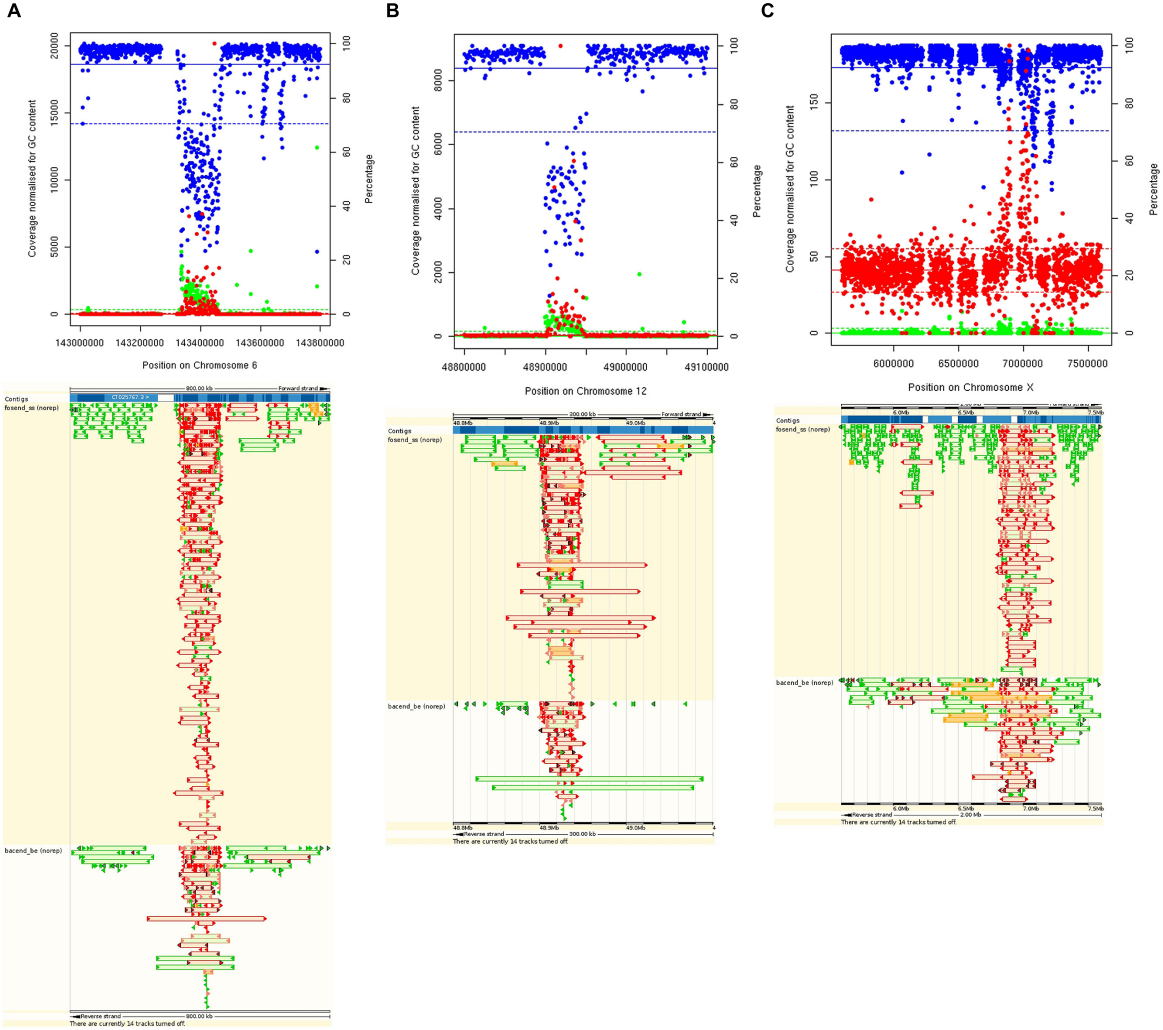
**Plots showing examples of abnormal regions for multiple parameters on Chromosomes 6 **(A)**, 12 **(B)**, and X **(C)** (top).** GC normalized coverage (red) uses the left *Y* axis. Percentage of properly paired reads (blue) and percentage of high insert sizes (green) use the right *Y* axis. Means are represented by solid lines and 2 *SD* from the mean are represented by dashed lines. Bottom shows same regions viewed on the gEVAL browser with poorly mapped fosmids (top) and bac ends (bottom) shown in red.

Regions identified by the parameters measured are summarized in **Table 1**. In total, 2.6% of the genome had abnormally high coverage, and 26.6% of the genome abnormally LC. Regions with a high percentage of fragment pairs with abnormally low and high insert sizes cover 3.99% and 1.52% of the genome, respectively. Regions with a low percentage of properly paired reads cover 4.95% of the genome. One of the largest regions identified (77.8 Kb) has abnormal coverage, insert sizes and read orientation (**Figure 2A**), and this is not uncommon, further examples are shown in **Figures 2B,C**.

**Table 1:** Table summarizing the regions identified by different parameters measured.

	No. of features	Mean feature size	Percentage of genome
High coverage	60,281	1,202	2.6
Small insert	82,097	1,363	3.99
Large insert	31,833	1,343	1.52
Improperly paired	77,785	1,786	4.95
Homozygous variants	245,972	256	2.25
Low quality (LQ)	409,905	949	13.85
Low coverage (LC)	119,251	6,275	26.6
**Total (LQLC)**	337,276	2,753	33.07

There were a total of 62,463 regions with >50% multimappers and of these 99.3% overlapped with the LC regions. 66% of the regions identified as LC overlapped with the multimapped regions. The remaining LC regions had an unremarkable distribution of GC contents (data not shown) and the majority (81%) had 0 multimappers. The median read count per window for the whole genome was 264 and the median read count per window for the LC regions excluding those with >50% multimappers was 161.

We identified a total of 583,093 homozygous variants. Following merging, there were 245,972 regions identified as abnormal due to proximity to these variants covering 63,085,828 bases (2.25% of the genome).

### Merged Regions

After merging the regions with abnormal insert sizes, abnormal read orientation, and homozygous variant calls, we were left with 409,905 regions identified as being LQ, covering 13.85% of the genome.

In total, 337,276 regions were identified as being LQLC and the regions covered a total of 928,664,896 bases (33.07% of the genome). If the multimapped regions are excluded from the LC regions and the remaining LC regions are merged with the LQ regions these cover 17.3% of the genome.

The coding region data downloaded from UCSC table browser covered 587,219,382 bases (excluding chromosome Y) and of these 81,566,904 (13.89%) intersected with the LQ regions.

Of the coding region, 154,875,678 bases (26.37%) intersected with the LQLC regions.

### Impact on Public Data

The proportion of variants from publicly available data sets from Paudel et al. (2013) and dbSNP (Sherry et al., 2001) that fall in the abnormal regions are summarized in **Table 2**.

**Table 2:** Table summarizing the proportion of called variants in publicly available data that fall in the abnormal regions identified in the current study.

	Total	LQ	LC	Combined (LQLC)
% of genome	–	13.85%	26.6%	33.07%
% of coding region	–	13.89%	17.72%	26.37%
dbSNP variants^a^	52,634,111	19,121,760 (36.33%)	15,483,445 (29.42%)	27,009,232 (51.3%)
CNVRs^b^	3,118	1,081 (34.66%)	1,706 (54.71%)	2,692 (86.3%)
RNA-seq genes^c^ (intersecting bases)	41,788,900	11,155,280 (26.69%)	11,360,980 (27.19%)	17,959,798 (42.98%)

*^a^Data from dbSNP database (Sherry et al., 2001).*

*^b^Data from Paudel et al. (2013).*

*^c^Data from Ensembl (Cunningham et al., 2015).*

Paudel et al. (2013) identified 61,761 multi-copy regions (MCR), and from these identified 3,118 CNVRs. Of the CNVRs 1,081 (34.66%) lie in the LQ regions and 2,692 (86.3%) lie in the LQLC regions identified here.

The data downloaded from dbSNP (Release 104. Accessed: 05/05/2015) contain 52,634,111 known variants. In total, 19,121,760 (36.33%) dbSNP variants were located in the LQ regions, 15,483,445 (29.42%) dbSNP variants were located in the LC regions and 27,009,232 (51.3%) dbSNP variants were located in the LQLC regions.

The gene predictions based on RNA-sequencing data covered 41,788,900 bases, 26.69% of these bases were in the LQ region (11,155,280), 27.19% were in the LC region (11,360,980) and 42.98% were in the LQLC regions (17,959,798).

## Discussion

This work emphasizes the importance of accuracy in reference genomes in variant discovery research. Previous work by Zhang and Backstrom (2014) used sequencing reads from multiple chickens to detect misassemblies in the chicken genome. Here we used data from the same individual used to construct the pig reference assembly. We are therefore able to assess the assembly without introducing potential true variation that may be present by chance in multiple individuals; however, regions of the genome may have been incorrectly identified as low-quality due to true structural variation at heterozygous sites.

Regions of Sscrofa10.2 identified in this study were enriched for variants from dbSNP. The fact that the regions identified were enriched for variants in dbSNP, with the LQLC regions containing over half of the dbSNP variants, supports the assertion that these regions are enriched for false-positives; dbSNP contains large numbers of SNPs that are not validated and are potentially false-positives (Mitchell et al., 2004; Musumeci et al., 2010).

In the CNVR study by Paudel et al. (2013), 61,761 MCRs were identified and the authors state that the majority of these were common in all individuals sequenced; in this study 60,281 regions were identified as having high coverage and it is likely that there is overlap between these results. Studies looking for copy number gains may benefit from excluding the LQ regions from analysis. From the MCRs, 3,118 CNVRs were identified. The authors estimated that of these 2,664 (85.43%) were likely to be neutral or nearly neutral as they were common between different groups or were in non-genic regions, which is very similar to the number of CNVRs in the data that overlap the LQLC regions in the current study (2,692; 86.3%). CNVRs are called from sequencing data by comparison of read counts for a region with the average across the genome; it is likely that there are many false tandem repeats or collapsed repetitive regions in the assembly that would cause false copy number loss or gain calls. While regions identified as CNVRs are potentially variable regions between populations, breeds and individuals, calls based solely on comparison with the reference will give false-positives and false estimates of the copy numbers in true variable regions. Paudel et al. (2013) used copy number comparisons between individuals from different populations to identify MCRs that were variable between groups, which likely removed the majority of the false-positives. Other studies have used array-based methods to detect CNVRs in the pig genome (Chen et al., 2012; Wang et al., 2012) and of the regions identified in these studies, almost all of them fall in the LQLC regions (data not shown). This suggests these regions truly are enriched for CNVRs; however, enrichment of the unreliable regions for CNVRs may also suggest unreliable assembly around large duplications. In studies using whole genome resequencing, often small sample sizes are used and too much confidence may be given to the reference. It would be advisable in studies using Sscrofa10.2, and references of other species that may contain similar inaccuracies, not to call CNVRs based solely on comparison with the reference, but from regional variation in read count between individuals as has been done previously for genomes which lack a reference following co-assembly (Nijkamp et al., 2012) and when comparing sequences from cancer cells to healthy cells (Chiang et al., 2009; Koboldt et al., 2012). Similarly, researchers using other techniques that rely on counting reads mapped to the reference genome such as ChiP-seq and RNA-seq should be aware that these errors may cause inaccurate calling or expression estimates. In RNA-seq, read counts are used to estimate expression levels; unexpected CNV between the reference and the sample sequence could cause over- or under-exaggerated read counts, potentially resulting in false-positives or false-negatives. RNA-seq is prone to off-target mapping (Mortazavi et al., 2008), particularly at higher depth (Tarazona et al., 2011); true peaks can often be distinguished from off-target mapping using an expression threshold. However, misrepresentation of the copy number of a region in the reference assembly may exaggerate off-target peaks above the threshold and cause false-positives, exaggerate true peaks causing inaccurate expression estimates, or reduce true peaks causing false-negatives or underestimation of expression. The regions identified here were enriched for RNA-seq gene predictions, more so than the annotated coding region, which may suggest an increased false-positive rate in these regions from this method.

A large amount of the genome showed LC. While these regions may suggest errors in the reference genome, such as false tandem duplications (Zhang and Backstrom, 2014), they do so with less confidence than the other parameters measured. The study by Paudel et al. (2013) reported a considerable number of copy number losses and subsequently excluded these from further analysis as they were likely enriched for false-positives; the fact that this excess of LC regions has been encountered by other researchers may suggest that the problem is with the quality of the genome assembly or region mappability rather than the quality of the data used in the current study. Regions with LC were analyzed separately as LC may be an indicator of the quality of the sequencing data, PCR bias or poor mappability and not necessarily inaccuracy in the reference. The majority of the LC regions were explained by their large proportion of multimappers; the regions were identified as LC because multimappers were excluded from the coverage analysis. These regions may not be misassembled, but rather of poor mappability due to, for example, low complexity or repetitive sequences. Of the LC regions which were not explained by multimappers there was no evidence of extreme GC content causing the reduced coverage and the majority contained no multimappers; the LC in these regions likely relates to misassembled areas in the reference genome, or potentially heterozygous structural variants in the individual. Where the LC is explained by poor mappability, it may still be advisable to exclude these regions from SNP and indel analyses as this is likely to yield LQ variants with a high rate of false-positives. Studies requiring identification of only the highest quality variants would reduce computational burden and false-positive rate by excluding the LC regions. In studies more concerned with finding variants relating to a specific phenotype, if LC regions are included, variants identified in them may be treated as low-confidence, but not necessarily excluded entirely. The percentage of dbSNP variants in the LC region is not as high as in the LQ region, however, fewer variants may be called in poor mappability regions due to the common practice of filtering out low mapping quality reads before proceeding to variant calling, reducing depth and subsequently the chances of calling a variant in these regions. The proportion of the genome identified here as LQ is likely to be an over-estimation of the proportion that is misassembled. The individual may have true, heterozygous structural variation that cause some of these regions to appear misassembled and this analysis has been intentionally strict to allow downstream bioinformatic analysis to focus on only the highest confidence regions of the genome by excluding LQLC regions. The number of variants identified in studies employing variant calling is often extreme and strict filtering techniques are employed to reduce the number to a more tractable level for validation (MacArthur et al., 2012; Ai et al., 2015). Excluding regions which are likely to be enriched for false-positives may significantly reduce computational burden and increase accuracy. Strict filtering after variant calling may cause the loss of variants of interest and it is desirable to reduce the initial number of variant calls as much as possible to reduce the need for excessive filtering. While variants of interest may lie in the low-confidence regions identified here, the excess of false-positives in the region make it unlikely that they will be easily identified. However, discovery of variants outside of these regions will benefit from the reduced number of false-positives in the dataset. Many variant callers and filtration methods will consider depth and mapping quality and are likely to exclude a number of false-positive variants from these regions by default; however, computational burden would be decreased by excluding unreliable regions, which will be particularly relevant with large datasets. Other methods that use regional read count data need to be aware that Sscrofa10.2 does not accurately represent the copy number of alleles in the original Duroc sow’s genome. Clearly in studies searching for CNVRs, excluding the LQLC regions, which are potentially enriched for true CNVRs, is not an option. In such studies it would be beneficial to compare individuals in a study with one another rather than with the reference, as is done in somatic variant calling comparisons between healthy cells and cancer cells (Roberts et al., 2013), to filter out variation that is common in all individuals, or to exclude the LQ regions only. The degree to which misassemblies will affect research results depends on a number of factors including the tools used, the type of misassembly and the type of analysis; for example, the incorrect order of contigs will negatively affect read-pair mapping and collapsed duplications may cause incorrect calling of SNPs – though SNP callers may accurately filter many of these. Similar inaccuracies to those found here are likely to be present in the reference genomes of other non-human species. With the price of sequencing continuing to fall, the number of large-scale sequencing studies on species with draft genomes will undoubtedly increase; awareness of inaccuracies in these references will decrease computational burden and increase accuracy. Identifying regions that are inaccurate and producing new, more accurate assemblies will greatly increase the power of whole-genome resequencing studies in non-human species.

### Availability of Data

The regions identified in this study have been made available as three bed files: LQ regions, LC regions, and LQLC regions. BED files are available to download from http://www.ark-genomics.org/outputs/identification-low-confidence-regions-pig-reference-genome-sscrofa102

## Conflict of Interest Statement

The authors declare that the research was conducted in the absence of any commercial or financial relationships that could be construed as a potential conflict of interest. The reviewer Martien Groenen declares that, despite collaborations with the author Alan L. Archibald, the review process was handled objectively.

## Footnotes

1 http://www.ebi.ac.uk/ena/data/view/ERP010190

2 http://sourceforge.net/p/picard/wiki/Main_Page/

3 http://geval.sanger.ac.uk/index.html
